# Substituting Fishmeal with Poultry By-Product Meal Enhances Economic Efficiency in Rainbow Trout (*Oncorhynchus mykiss*) Farming

**DOI:** 10.3390/ani15182723

**Published:** 2025-09-17

**Authors:** Víctor Jesús Vergara-Rubín, Víctor Rodrigo Guevara-Carrasco, Brenda Yance-Flores, Andrea Marchán-Timorán, Percy Bustamante-Gonzales

**Affiliations:** 1 Laboratorio de Investigación en Nutrición y Alimentación en Peces y Crustáceos (LINAPC), Departamento de Nutrición, Universidad Nacional Agraria La Molina, Lima 15024, Peru; 2 Programa de Investigación y Proyección Social en Alimentos (PIPSA), Departamento de Nutrición, Universidad Nacional Agraria La Molina, Lima 15024, Peru; 3 Department of Nutrition, Faculty of Animal Science, Universidad Nacional Agraria La Molina, Lima 15024, Perubyance@lamolina.edu.pe (B.Y.-F.); amarchan@lamolina.edu.pe (A.M.-T.); pbustamante@lamolina.edu.pe (P.B.-G.)

**Keywords:** *Oncorhynchus mykiss*, trout, poultry by-product

## Abstract

The global aquaculture industry faces increasing pressures to find sustainable and economically viable alternatives to traditional fishmeal, a key component in aquafeeds. This study investigates the economic impact of replacing fishmeal with poultry by-product meal (PBM) in large-scale farming of rainbow trout (*Oncorhynchus mykiss*), focusing on the Economic Conversion Ratio (ECR) as a primary metric. Conducted at the Sociedad Agrícola de Interés Social Túpac Amaru in Peru, the research utilized a multi-phase experimental design with a total of 46,320 fish. In the first phase, fish with an average initial weight of 4.34 ± 0.32 g were distributed across 12 concrete experimental units (2.25 m^3^ effective capacity each), with 4 replicates per treatment. Fish were initially fed a diet with a 5% fishmeal replacement, progressing to 30% and 45% replacements in subsequent phases, with specific fish selections and numbers for each stage. Water quality was meticulously monitored throughout the study to ensure optimal conditions. Key performance metrics, including initial and final body weights, weight gain, feed intake, and feed conversion ratio, were measured alongside economic parameters such as ECR. Results indicated no significant differences in growth performance across all phases, except for ECR. The commercial diet consistently demonstrated a lower ECR compared to PBM-inclusive diets, suggesting a higher cost-efficiency. This study concludes that poultry by-product meal (PBM) can effectively serve as a partial substitute for fishmeal, specifically up to 45% inclusion, without compromising the growth performance of rainbow trout. However, its overall economic viability still requires careful consideration of feed costs relative to fish sale prices.

## Simple Summary

The global fish farming industry is looking for cost-effective and sustainable alternatives to fishmeal, a common ingredient in fish feeds. This study was carried out to find out whether replacing fishmeal with poultry by-product meal (PBM) in the diet of farmed rainbow trout could be both practical and economical. The research was carried out on a commercial fish farm in Peru where three different diets using an increasing proportion of PBM were tested. Fish exhibited similar growth performance on PBM-based diets and traditional fishmeal; however, the economic analysis indicated that while PBM proved more cost-effective during periods of rapid growth, it was not the most economical option across the entire production cycle. This highlights the importance of carefully balancing feed costs with fish market prices before replacing fishmeal on a large scale. These findings will help fish farmers and the aquaculture industry make informed decisions about sustainable feed sources while still maintaining profitability.

## 1. Introduction

The rapid growth of aquaculture has driven an increasing demand for sustainable and cost-effective feed alternatives to fishmeal, the primary protein source in trout farming. Fishmeal production is becoming increasingly expensive due to overfishing, environmental concerns, and market fluctuations, necessitating the exploration of alternative protein sources that can maintain high growth performance without compromising fish health or final product quality [1,2,3,4].

Poultry by-product meal (PBM) has emerged as a promising alternative due to its high digestibility, balanced amino acid profile, and lower cost compared to fishmeal [5]. Several laboratory studies have investigated PBM’s effectiveness in replacing fishmeal in aquafeeds, yielding mixed but largely positive results. For instance, a study using rainbow trout with an average initial weight of 50.6 ± 1.35 g found that up to 75% of fishmeal protein could be replaced with PBM without negatively affecting growth performance. However, full replacement in diets formulated with 43% crude protein and 13.28 MJ DE kg^−1^ resulted in lower weight gain [4]. Another experiment, conducted with juveniles of rainbow trout (average initial weight 50 ± 0.42 g) over two months, formulated four experimental diets with PBM replacing 0, 33, 66, and 100% of fishmeal. This study showed that while replacing 33% of fishmeal with PBM was optimal, higher replacement levels (66% and 100%) led to increased liver fat accumulation and lower weight gain, indicating potential health risks [6]. Conversely, a study conducted a four-month feeding trial on rainbow trout with an initial stocking density of 20 fish/m^2^ and initial average weight of 122.5 ± 22.5 g, found that complete replacement of fish meal with a poultry by-product based diet resulted in no significant differences in growth performances (including weight gain, specific growth rate, biomass production, mean daily feed intake, feed conversion ratio, and survival rate) compared to a commercial diet [2].

The observed variability in research findings regarding PBM’s efficacy can largely be attributed to extensive studies highlighting the diverse nutritional content of rendered animal protein ingredients. This variability stems from differences in the quality and freshness of the raw poultry by-products, as well as the specific processing techniques employed during meal production. Consequently, pet food grade poultry meal is being proposed by rendering companies as a higher-quality alternative, capable of yielding more consistent and improved production results in aquaculture.

Despite these numerous promising laboratory findings, there remains a critical knowledge gap concerning the practical efficacy and economic viability of PBM in large-scale, commercial trout farming operations. While controlled conditions have demonstrated that PBM can support similar feed conversion ratios and weight gain as fishmeal-based diets [7], translating these results to a commercial scale introduces complex variables. These include practical feed manufacturing considerations, the nuances of water quality management in larger systems, and crucially, the overall economic feasibility and market acceptance of such dietary shifts. These factors are often not fully captured in laboratory settings and are essential for widespread industry adoption.

Therefore, to scientifically inform the optimization of trout feed formulations for improved cost-efficiency and to foster a more sustainable and economically viable aquaculture sector that can significantly enhance protein supply for the Peruvian population, particularly in high-altitude regions, comprehensive large-scale evaluations are indispensable. Hence, this study aimed to evaluate the effect of replacing fishmeal with poultry by-product meal on the growth performance and economic feasibility (Economic Conversion Ratio) of rainbow trout (*Oncorhynchus mykiss*) in a large-scale commercial farming setting in Peru.

## 2. Materials and Methods

The present research was made ethically according to animal care and protection principles of Peruvian National Laws. The experimental protocol was approved by the Ethics Committee of Universidad Nacional Agraria La Molina on 30 November 2022 (05-2022-CEI-UNALM). Although researchers were not blinded to the treatment allocation, data collection and analysis were conducted using standardized protocols to minimize potential bias.

### 2.1. Digestibility Trial

The evaluations were carried out at the Laboratory of Nutrition and Feeding Research for Fish and Crustaceans (LINAPC) of the Academic Department of Nutrition, Faculty of Zootechnics, Universidad Nacional Agraria La Molina. The experimental diets were manufactured at the Animal Feed Plant of the Food Research and Social Outreach Program, Faculty of Zootechnics.

The Laboratory (LINAPC) is equipped with aquariums located under a water recirculation system. Two acclimatization aquariums of 120 L each, 18 aquariums of 75 L for growth trials and nine aquariums of 55 L Guelph type for digestibility trials. Water was recirculated through mechanical (and biological filters (inoculated with aerobic, anaerobic, and facultative bacteria), continuous aeration was provided (1 hp air blower) and temperature maintained (heat pump 12,000 BTU/h). Water parameters were kept according to the requirements of the species; temperature (10–12 T°) and dissolved oxygen (5.48 mg/L) were recorded daily, while Ph (7.13), water hardness (145 ppm), total ammonia–nitrogen (0.21 mg/L) and nitrite (0.20 mg/L) were measured three times a week. Temperature, dissolved oxygen and Ph were measured with a Multi-parameter (model HI9829, Hanna Instruments, Woonsocket, RI, USA), GH test kit was used to measure water hardness and a colorimetric kit PRO AQUATES was used to measure total ammonia nitrogen and nitrite.

#### 2.1.1. Fish, Feeding, Experimental Diets

An in vivo digestibility test was conducted to evaluate the apparent digestibility coefficients (ADC) of the nutrients and digestible energy of the ingredients. The trial was carried out in the LINAPC digestibility tanks, where 45 trout with an average weight of 215.24 g and an average size of 25.70 cm were randomly distributed in 9 aquariums.

The experimental diets consisted of 69.5% of the reference diet, 30% of the test ingredient and 0.5% of chromium oxide (Cr_2_O_3_) as an external inert marker. Ingredients were grounded with a disk mill (100 µm average), weighed, mixed and extruded. All diets were manufactured using a twin screw extruder (model DRX-300 CH, Food and Machines, Ribeirão Preto, SP, Brazil). Final moisture levels were less than 10%. All oil was included in the mix rather than top coated. Representative samples of the diets were sent to the laboratory to determine the proximal nutrient content (Table 1).

Poultry by-product (PBP) used in this research comes from a local rendering company. It is composed of necks, feet, heads, viscera and lean tissues. These residues were cooked in its own moisture and fat with dry heat in open steam jacketed drums until the moisture evaporated (133 °C, 3 bar, 20 min). After dehydration, as much fat as possible was removed by draining and the residue was passed through a screw press inside a closed vessel to remove some of the remaining fat and moisture. After cooking and fat separation, the cracklings, which contain protein, minerals and some residual fat, continue in the process to remove additional moisture. Finally, it undergoes a grinding process in a hammer mill to achieve the desired particle size.

For the first week after stocking, fish were fed two times daily until apparent satiation with the experimental diets without collecting feces to adapt the organisms to feeding and handling practices. After this adaptation period, aquariums and the collection system were cleaned to start the feces collection. Fish were fed at 8 h and 17 h and feces were collected daily in the morning and afternoon before feeding. The collected feces were placed in glass plates oven dried (65 °C) and stored −4 °C for subsequent chemical analysis. Feces collection was performed over a 42-day period and the samples were analyzed separately for each replicate. No mortality or disease signs occurred during the acclimation and experimental period.

Chemical analyzes were performed in external laboratories. The proximate composition of the ingredients, experimental diets and feces were analyzed according to standard methods [8] and Gross energy (GE) were determined in a calorimetric bomb following the ASTM D2015-66 standard [9]. Chromic oxide in ingredients and feces were analyzed using inductively coupled plasma optical emission spectroscopy technique (ICP-OES). The chemical composition of the ingredients is shown in Table 2.

#### 2.1.2. Calculations

Apparent digestibility coefficients (ADC) for protein, ether extract and gross energy of the reference and test diets were calculated using the formulas described in [10,11].

$${ADC}_{\left(d\right)}=100-\left(100\left(\frac{\%{Cr}_{2}O_{3(d)}}{\%{Cr}_{2}O_{3(h)}}\right)\times \left(\frac{\%{Nut}_{\left(h\right)}}{\%{Nut}_{\left(d\right)}}\right)\right)$$

where: ADC (d) = apparent digestibility coefficient of the reference and test diet, Cr_2_O_3_(d) = % chromium oxide in the diets, Cr_2_O_3_(h) = % chromium oxide in the feces, Nut(d) = % of the nutrient in the diets and Nut(h) = % of the nutrient in the feces. Then the ADCs of the ingredients were determined with the following formula:

$${CDA}_{\left(ing\right)}={CDA}_{\left(dp\right)}+({CDA}_{\left(dp\right)}-{CDA}_{\left(dr\right)})\times (\frac{b\;*\;N_{\left(dr\right)}}{a\;*\;N_{\left(i\right)}})$$

where: CDA(ing) = apparent digestibility coefficient of the ingredient, CDA(dp) = apparent digestibility coefficient of the test diet, CDA(dr) = apparent digestibility coefficient of the reference diet, a = percentage of the test ingredient, b = percentage of the reference diet, N(dr) = percentage of nutrients or gross energy of the reference diet and N(i) = percentage of the ingredient or gross energy of the test ingredient. And finally the digestible energy content was determined from the following formula:

$${{ED}_{\left(ing\right)}=CDA}_{\left(ing\right)}\times {EB}_{\left(ing\right)}$$

where: ED(ing) = digestible energy of the test ingredient, CDA(ing) = apparent digestibility coefficient of the test ingredient and EB(ing) = gross energy of the ingredient.

### 2.2. Growth Trial Large-Scale

The evaluation was conducted at Sociedad Agrícola de Interés Social Túpac Amaru (SAIS), a Peruvian agricultural organization located in the District of Canchayllo, Province of Jauja, Department of Junín. The organization’s headquarters are at Hacienda Pachacayo, situated at kilometer 43 of the central La Oroya-Huancayo highway. SAIS manages over 200,000 hectares across diverse ecosystems in the central Andes. The experimental units were housed in the Vinchos Reproduction Sub-Unit, which sits at an altitude of 3771 m above sea level. The unit’s water supply originates from Elena Puquio, a natural spring.

The experimental units were arranged in parallel, constructed of concrete, and had an effective capacity of 2.25 m^3^ with an average water flow rate of 1.25 L/s. Water quality parameters were regularly monitored. Temperature (10–12 °C) and dissolved oxygen (6.8–7.09 mg/L) were recorded daily, while pH (7.05), water hardness (145 ppm), total ammonia nitrogen (0.05 mg/L), and nitrate (0.09 mg/L) were measured three times per week. Measurements were taken using a HANNA HI9829 multiparameter device (Hanna Instruments, Woonsocket, RI, USA) for temperature, dissolved oxygen, and pH. Water hardness was assessed using a GH test kit, while total ammonia nitrogen and nitrate levels were determined with a PRO AQUATES colorimetric kit.

#### 2.2.1. Fish, Feed Ingredients and Experimental Diets

A total of 46,320 fish, with an average initial weight of 4.34 ± 0.32 g, were distributed across 12 experimental units. The units were divided into three groups: four were fed a control diet without PBM inclusion, another four received diets containing PBM, and the remaining four were fed a commercially balanced diet (Table 3). The chemical composition and market price of the ingredients is shown in Table 4 and the formulations of these diets are detailed in Table 5. In the first stage, the fish were fed a diet with a 7.5% fishmeal replacement for 45 days. At the end of this period, a biometry assessment was conducted, and the fish were classified by size into small and large categories. For the second stage, 1150 fish selected from the large-size category of the previous phase were used, with an average weight of 24.28 ± 0.89 g. These fish were fed a second-stage diet containing a 30% fishmeal replacement with PBM for 39 days. At the end of this phase, another biometry assessment and size-based selection were performed. 400 fish with an average weight of 63.93 ± 2.93 g were selected. They were fed the same diet with a 30% fishmeal replacement for 30 days but with a bigger feed diameter. Finally, 400 fish from this stage were selected and fed for an additional 35 days with a diet containing a 45% replacement of fishmeal with PBM. All experimental diets were formulated meeting the nutritional requirements for rainbow trout at different life stages, as recommended by National Research Council [10]. The diets were primarily formulated to be iso-nitrogenous and iso-energetic, targeting specific levels of crude protein and gross energy appropriate for each growth phase. While these primary nutrient targets were consistently maintained, minor differences in the composition of other nutrients occurred as a result of accommodating the varying inclusion levels of poultry by-product meal. The duration of each feeding phase (45, 39, 30, and 35 days, respectively) was specifically designed to align with typical commercial production cycles and the industry practice of size-based grading in high-altitude trout farms. In these systems, fish are regularly harvested and re-distributed based on their size to optimize tank utilization and growth efficiency, leading to shorter, distinct feeding periods for specific size classes rather than a single, continuous grow-out. While longer experimental durations are beneficial for detecting subtle long-term physiological or metabolic changes, our study’s primary objective was to evaluate the practical growth performance and economic feasibility of PBM diets under these real-world commercial production conditions.

The experimental diets were formulated to gradually increase the inclusion of poultry by-product meal (PBM) across the three growth phases, which corresponded to increasing fish size and developmental stage. Specifically, PBM was included at 5%, 18%, and 20% in the first, second, and third phases, respectively. These PBM inclusion levels translated to a fishmeal replacement of approximately 7.5%, 30%, and 45% in the respective phases. This progressive replacement strategy was chosen based on two key considerations. Firstly, a lower initial PBM inclusion (5%) was implemented in the first phase because younger, smaller fish are generally more sensitive to novel ingredients and dietary changes, and their digestive systems may be less developed. This cautious approach aimed to minimize any potential negative impacts on growth or health during this critical early growth period, when commercial mortality rates are typically higher. Secondly, as fish grow larger in subsequent phases, their physiological robustness and ability to efficiently utilize alternative protein sources generally increase. This allowed for higher PBM inclusion levels (18% and 20%) in the later phases, aiming to maximize fishmeal replacement and achieve greater economic benefits without compromising performance as the fish approached market size.

#### 2.2.2. Biochemical Analysis

Chemical analyzes were performed in external laboratories. The proximate composition of the ingredients and experimental diets were analyzed according to standard methods Official Methods of Analysis of the Association of Official Analytical Chemists [8] and Gross energy (GE) was estimated using the caloric values of protein, fat, and carbohydrates. Four fish from each experimental unit at the end of the trial were randomly sacrificed by a lethal bath of clove oil (150 mg L^−1^) and pooled for whole-body composition analysis. The sampled fish were dried at 105 °C. Contents of dry matter, crude protein, crude lipid and ash were determined following the AOAC procedures [12]. 

### 2.3. Calculation and Statistics

Feed intake, weight gain, feed conversion ratio (FCR) and feed cost were calculated as below:

Feed intake (g/fish) = dry feed intake/number of fish.

Feed intake was determined daily by subtracting the weight of uneaten, dried feed from the initial amount of feed offered. The total dry feed intake for each tank was then divided by the number of fish in that tank to obtain the feed intake (g/fish)

Weight gain (g) = Wt − W0

FCR = I/(Wt − W0)

where I(g) is the total amount of offered feed, Wt is the weight of the fish at the end of trial (g), and Wo is the weight of fish at the beginning of the trial (g).

Economic conversion ratio (ECR, USD/kg) = Feed consumption (kg/fish) × diet price (USD/kg)/weight gain of fish (kg/fish).

The data were expressed as the mean ± SD, and statistical analyses were performed using the SAS^®^ OnDemand for Academics (SAS ODA) web application, version 9.4 (SAS Institute Inc., Cary, NC, USA). Statistical analysis of data was done by one-way analysis of variance (ANOVA) with 0.05 as probability level for rejection of the null-hypothesis. Duncan test was used to assess significant differences among means. Statistical assumptions, including normality, were checked. The Shapiro-Wilk test was used to assess normality, Bartlett’s test was employed to confirm the homogeneity of variances and to check for the independence of errors, the residuals were plotted.

## 3. Results

### 3.1. Digestibility Trial

The apparent digestibility coefficients (ADC) of nutrients and energy for each ingredient are shown in Table 2. The results of ADCs of dry matter for PBM reported by other authors [13] ranged from 70.9 to 74.5%, which is closer to our results (68.59). However, higher ADCs of dry matter (92%) were reported in another study [14]. The ADCs of crude protein were found to be 78.92%, lower than the values presented in previous research [13,14], which reported ranges of 83.1–87.1% and 88%, respectively. Similar values to ours (64.4–77.7%) were observed in another study [15]. ADCs of lipid were lower than those reported in previous research [13,16], which found values of 79.7–82.7% and 92%, respectively. Gross energy digestibility of PBM was higher than the 88% and 87% reported in earlier studies [13,16]. The studies mentioned used different protocols, including variations in collection methods and inert indicators; however, research [17] suggests that these methodological differences do not significantly impact results. This supports the idea that the observed differences are primarily due to ingredient variability. Although the PBM used in this study was pet food-grade, its quality was not guaranteed, as reflected in the digestibility results.

### 3.2. Growth Trial Large-Scale

The present study evaluated the economic impact of substituting fishmeal with poultry by-product meal (PBM) in large-scale rainbow trout farming across three experimental phases. The results from the first phase, detailed in Table 6, revealed no significant differences in initial body weight (IBW), final body weight (FBW), weight gain (WG), feed intake (FI), and feed conversion ratio (FCR) across the different dietary treatments. Similarly, the size-based categorization of fish into large and small groups did not yield any significant differences in these performance metrics. However, a notable finding was observed in the Economic Conversion Ratio (ECR), where diets incorporating 5% PBM exhibited a higher ECR compared to commercial diets, which demonstrated the lowest ECR values. The control diet, devoid of PBM inclusion, showed the highest ECR, indicating less economic efficiency. Additional data on the growth performance of the trout are provided in the Supplementary Materials (Table S1).

In the second phase, as presented in Table 7, the performance parameters—IBW, FBW, WG, FI, and FCR—continued to show no significant differences across the dietary treatments. A distinct pattern emerged concerning the size distribution of the fish; those fed with commercial diets had a higher proportion of larger individuals compared to those on control diets or diets with fishmeal replaced by PBM. This suggests a potential influence of diet composition on growth variability within the population. The ECR analysis in this phase further highlighted significant differences, with the control diet consistently exhibiting higher ECR values, reaffirming its relative economic inefficiency. Despite the reformulation of diets for larger-sized fish in this phase, no significant differences were detected in the performance or economic parameters post-selection.

The third phase, summarized in Table 8, continued the trend of non-significant differences in performance metrics across dietary treatments. However, a significant outcome was observed in the ECR, where the value was notably lower for diets containing PBM. This suggests an improvement in economic efficiency when PBM is utilized at higher inclusion rates. This phase’s results underscore the potential economic benefits of PBM, as its inclusion consistently resulted in a more favorable ECR compared to the control diet.

Taken together, our results indicate that while the substitution of fishmeal with poultry by-product meal does not adversely affect the growth performance of rainbow trout, it significantly enhances economic efficiency, as evidenced by a consistently lower Economic Conversion Ratio. The findings of this study indicate that there were no significant differences in body composition between experimental groups, except for ash content, which was significantly higher in fish fed diets containing poultry by-product meal (PBM). Proximate composition of trout is shown in Table 9. These results align partially with those reported by [18], who found that carcass ash levels did not significantly differ at a 50% PBM replacement level, though higher PBM levels influenced moisture and lipid content. Similarly, the present study showed no significant variation in lipid content, consistent with the findings of [19], who also reported stable lipid levels across different PBM dietary treatments. However, while [19] observed no significant changes in ash content among dietary groups, the current study demonstrated a notable increase in ash levels in PBM-fed fish, suggesting a possible variation in PBM composition or processing. Furthermore, crude protein levels remained unchanged in this study, a result more in line with [19] than with [18], who reported a significant reduction in protein content in PBM-fed fish.

## 4. Discussion

The aquaculture sector, responsible for about half of the world’s fish supply, has faced growing pressure to reduce reliance on conventional feed components like fishmeal, for the sake of sustainability. Fishmeal is nutritionally optimal but faces economic volatility and environmental sustainability issues due to overfishing [20]. This context has driven research on alternative protein sources that can sustain or increase aquaculture productivity without compromising environmental impact [21]. Poultry by-product meal (PBM) is a potential candidate, which represents a sustainable source of protein at an environmentally friendly cost as a substitute for fishmeal. PBM is gained from the processing of poultry waste and thus helps to not only solve waste management issues but also serves as a high protein feed ingredient. The present study explores the economic impact of replacing fishmeal with PBM in large-scale rainbow trout production, and thus places emphasis on the Economic Conversion Ratio (ECR), a relevant feed cost efficiency measurement.

The main findings of the study, which came from a three-phase experimental design, were that PBM could be used to substitute some of the fishmeal in the diet of the rainbow trout. Notably, within the experimental phases there were no significant differences observed for growth performance metrics Initial body weight (IBW), Final body weight (FBW), weight gain (WG), feed intake (FI) and feed conversion ratio (FCR). Such similarity supports the nutritional equivalency of diets containing PBM in terms of supporting rainbow trout growth. However, the arguably most surprising finding relates to the ECR, which showed significant differences. In the first phase, these commercial diets had a lower ECR than the 5% PBM diets (the control diet tended to have the highest ECR). This pattern continued into the second phase, where once more commercial diets were more economically efficient, and it was even more marked in the larger fish. The third phase further corroborated these findings, with PBM-inclusive diets achieving a significantly lower ECR, thus highlighting their economic advantage over traditional fishmeal-based diets.

These findings are especially new as they provide empirical information on the economic advantages of PBM within large-scale aquaculture, an area that has been relatively neglected in literature. Although former researches have mainly examined the nutritional components of PBM, this study is the first to analyze the economic performance and economic efficiency in detail. These results suggest that ECR could be decreased with inclusion of PBM and PBM could represent an effective and cheaper alternative to fishmeal, as this ingredient maintains the sustainability of aquaculture systems. This supports the larger aspirations of the industry to lessen dependence on fishmeal, thus alleviating the environmental and economic pressures that come with its production.

Several theoretical and contextual reasons can account for the observed outcomes. PBM has a good nutritional profile as it is rich in proteins and essential amino acids, which explains the almost similar growth performance to fishmeal-based diets, as demonstrated by the IBW, FBW, WG, FI and FCR during the different phases of this study [22]. The cost reduction of the ECR could even be further increased by the lower cost of PBM compared to fishmeal. Finally, being produced in existing poultry processing facilities, PBM production can be scaled up easily to satisfy the protein requirements of many large-scale aquaculture producers. Taken together, these factors underpin the potential for PBM as a sustainable feed ingredient consistent with the industry’s transition towards more sustainable environmental and economic practices.

It is important to acknowledge the fatty acid profile of the experimental diets, particularly the reliance on soybean oil as the primary lipid source. While economically viable, soybean oil lacks the long-chain marine fatty acids (e.g., EPA and DHA) typically found in fish oil, which are crucial for optimal fish health, immune function, and flesh quality in salmonids like trout. Although no significant differences in growth performance were observed in this study, the absence of these specific fatty acids could potentially influence other physiological parameters or product quality attributes not measured herein. Future studies could explore the impact of dietary supplementation with marine or alternative sustainable sources of long-chain fatty acids on these aspects, even in diets formulated for economic efficiency.

The findings of this study are corroborated by previous research that supports the nutritional adequacy of PBM in aquaculture diets. Studies have demonstrated that PBM can replace a significant portion of fishmeal without compromising growth performance in various fish species, including tilapia and catfish [23,24,25,26]. These studies, like the current research, report comparable growth metrics and feed conversion ratios between PBM and fishmeal-based diets. The consistency of these findings across different species and experimental conditions reinforces the validity of PBM as a viable fishmeal substitute. Furthermore, the economic benefits observed in this study align with reports of cost savings associated with PBM-inclusive diets, highlighting its potential to enhance the profitability of aquaculture operations.

However, the current study also contributes to the discourse by addressing discrepancies with previous contradictory studies. Some research has reported potential limitations of PBM, such as variable digestibility and amino acid imbalances, which could affect growth performance [27,28]. These studies often emphasize the need for careful formulation and supplementation to optimize the nutritional profile of PBM-inclusive diets. The current study, by demonstrating consistent growth performance and economic advantages, suggests that these challenges can be effectively managed in large-scale operations. The findings highlight the importance of rigorous diet formulation and quality control in realizing the full potential of PBM as a fishmeal substitute. This contribution is particularly novel, as it provides a pathway to overcome the limitations identified in previous studies, thereby advancing the understanding of PBM’s role in sustainable aquaculture.

Although the conclusions may be reliable, the study acknowledges shortcomings that could affect how results are seen and applied. The experimental design, however comprehensive, operated within a specific ecological and economic context. Its findings can therefore be applied only to other regions or species with caution (e.g., those within similar contexts). Moreover, the study itself was greatly concerned with the econometric aspect of including PBM. How it affects fish health and product quality over the long term remains to be investigated. Overcoming these limitations in future research will be essential to realizing PBM as a sustainable feed ingredient in aquaculture.

By emphasizing the economic effect of experiments on big-scale rainbow trout farming, this work provides good advice towards aquaculture practices that sustainability can live with. This report offers the industry alternatives protein sources to fishmeal. The advent of PBM can make aquaculture much more sustainable for ecology and finance. This research not only deepens understanding of PBM in aquaculture but also feeds into policy and decision-making. The adoption of sustainable feeding practices is thus being advocated throughout today’s global aquaculture industry.

While the economic advantages of utilizing PBM are significant, particularly given our findings of no significant negative impact on growth performance or survival with the chosen replacement level, it is crucial to consider the broader implications. Although our study did not reveal detrimental effects on short-term health indicators, previous research [6] has indicated potential trade-offs, such as increased liver fat accumulation at higher PBM inclusion levels, suggesting a need for a balanced perspective. Therefore, future investigations should focus on long-term feeding trials to thoroughly evaluate any potential impacts on fish health to ensure sustainable and consumer-acceptable aquaculture practices. This will provide a more comprehensive understanding of the balance between economic benefits and biological outcomes when integrating PBM into aquafeeds.

Beyond the significant economic advantages, the utilization of poultry by-product meal (PBM) in aquaculture feeds aligns strongly with broader sustainability goals and offers notable environmental benefits. By incorporating PBM, we effectively value a co-product of the poultry industry, transforming what would otherwise be a waste stream into a valuable feed ingredient. This approach reduces waste disposal burdens and improves resource efficiency within the terrestrial animal production sector. More importantly, the replacement of traditional fishmeal with PBM directly contributes to mitigating the environmental impact of aquaculture. Fishmeal production is often associated with considerable ecological footprints, including pressures on wild fish stocks, bycatch, and energy consumption linked to capture fisheries and processing. By reducing reliance on fishmeal, PBM use can significantly lower the carbon footprint of aquaculture systems by decreasing the demand for wild-caught fish, lessening fuel consumption for fishing vessels, and minimizing energy inputs for fishmeal manufacturing. Thus, integrating PBM promotes a more circular economy within the food production chain, enhancing the overall environmental sustainability of farmed fish production and reducing its impact on marine ecosystems.

## 5. Conclusions

In summary, this study demonstrates that replacing fishmeal with poultry by-product meal (PBM) in rainbow trout diets offers significant economic benefits through improved cost efficiency, without negatively impacting growth performance. These findings highlight PBM’s potential as a practical, sustainable, and cost-effective alternative for large-scale aquaculture. While our results showed no adverse effects on short-term growth or survival, broader implications for fish health and product quality, as suggested by some previous studies, warrant further investigation. Therefore, future research should focus on long-term trials to fully assess the physiological health and marketable product quality of fish fed PBM-inclusive diets. Such studies will be crucial for validating PBM’s global applicability and ensuring the long-term sustainability and resilience of the aquaculture industry.

## Figures and Tables

**Table 1:** Composition of the reference diet.

**Ingredients**	**RD ^1^**
Fish meal, 66	55.72
Rice meal	25.54
Soybean meal, 47	9.95
Soybean oil	7.96
Growth promoter ^3^	0.20
Aquaculture premix ^2^	0.20
Choline Chloride	0.10
Antioxidant ^3^	0.03
Chromic oxide	0.50
**Nutrient**	**Proximal Composition**
Dry matter (%)	92.39
Crude protein (%)	46.70
Ethereal extract (%)	11.32
Ash (%)	10.71
Carbohydrate (%)	28.66
Gross energy (Mcal/Kg)	4.526

^1^ RD: Reference diet. ^2^ Vitamins (mg/kg diet). A: 4667 UI; D3: 667 UI; E: 47 UI; Choline chloride: 280 200; Niacin: 50; Riboflavin: 6.67; Tiamin: 6; Biotin: 0.27; Folic acid: 1.33; B12: 281 0.01; ascorbic acid: 200. Minerals (mg/kg diet). Manganesium: 13.33; Iodine: 282 0.50; Copper: 0.50; Zinc: 6.67; Iron: 6.67; Cobalt: 0.50 283. ^3^ Commercial products, Growth promoter: mano-oligosaccharide, Antioxidant: butylhydroxytoluene.

**Table 2:** Chemical composition and Apparent Digestibility Coefficients (ADC) of Dry Matter, Crude Protein, Lipid, Gross Energy (GE) and Digestible Energy (DE) of the Ingredients fed to “trout” (*Oncorhynchus mykiss*).

Ingredient	Chemical Composition				ADC				
	DM (%)	CP (%)	E.E * (%)	GE (Mcal/kg)	DM	CP	E.E * (%)	GE	DE (Mcal/kg)
Fishmeal	92.37	66.78	8.00	4.46	79.89	91.05	87.33	89.70	4.39
Poultry by-product	92.80	56.68	15.50	5.29	68.09	78.92	59.31	81.12	4.65

* Ethereal extract.

**Table 3:** Chemical compositions of commercial diets.

Nutrient	Proximal Composition		
	First Phase	Second Phase	Third Phase
Dry matter (%)	91.78	93.29	92.26
Crude protein (%)	47.36	48.89	40.54
Ethereal extract (%)	8.2	8.36	14.04
Crude fiber (%)	0.92	0.84	1.24
Ash (%)	11.59	11.4	7.96
Nitrogen-free extract (%)	23.71	23.8	28.48
Gross energy (Mcal/Kg)	4.47	4.57	4.85
Price (USD/kg) ^1^	2.34	2.16	1.95

^1^ Price: USD 1 = PEN 3.70.

**Table 4:** Proximate composition (g/kg) and market price (US$/kg) of the feed ingredients.

Ingredients	Dry Matter	Crude Protein	E.E ^1^	Ash	Price ^2^
Fish meal, 66	92.37	64.78	2.2	17.59	2.27
Poultry by-product	92.8	56.68	15.50	11.58	1.13
Rice meal	91.9	9.9	1.4	5.5	0.41
Soybean meal, 47	89.6	47.7	1.9	6.9	0.65

^1^ Ethereal extract; ^2^ Price of feed ingredients is calculated with the exchange rate of US$ to PEN at 3.70.

**Table 5:** Ingredient and chemical compositions of the experimental diets.

	First Phase		Second Phase		Third Phase	
Ingredients	Control	PBM 5%	Control	PBM 18%	Control	PBM 20%
Fish meal, 66	60	55.5	60	42	40	22
Poultry by-product	0	5	0	18	0	20
Rice meal	13.2	12.7	13.97	12.77	25.27	24.77
Soybean meal, 47	16	16	16	18.5	25	25
Soybean oil	9.5	9.5	9.5	8.2	9	7.5
Oxytetracycline	0.7	0.7	-	-	-	-
Salt	-	-	-	-	0.4	0.4
Aquaculture premix ^1^	0.2	0.2	0.2	0.2	0.2	0.2
Prebiotic^2^	0.2	0.2	0.2	0.2	-	-
Choline Chloride	0.1	0.1	0.1	0.1	0.1	0.1
Vitamine C	0.07	0.07	-	-	-	-
Antioxidant ^2^	0.03	0.03	0.03	0.03	0.03	0.03
**Nutrient**	**Proximal composition**					
Dry matter (%)	89.93	92.74	93.9	93.86	91.39	91.8
Crude protein (%)	48.81	48.37	48.57	48.53	38.77	38.61
Ethereal extract (%)	12.09	8.75	12.39	12.73	9.95	12.18
Crude fiber (%)	0.98	0.8	0.7	1.18	1.08	1.08
Ash (%)	12.11	11.49	11.41	10.68	9.5	7.94
Nitrogen-free extract (%)	15.94	23.33	20.83	20.74	32.09	31.99
Gross energy (Mcal/Kg)	4.59	4.55	4.80	4.85	4.50	4.70
Price (USD/kg) ^3^	2.61	2.56	2.42	2.17	1.99	1.74

^1^ Vitamins (mg/kg diet). A: 4667 UI; D3: 667 UI; E: 47 UI; Choline chloride: 280 200; Niacin: 50; Riboflavin: 6.67; Tiamin: 6; Biotin: 0.27; Folic acid: 1.33; B12: 281 0.01; ascorbic acid: 200. Minerals (mg/kg diet). Manganesium: 13.33; Iodine: 282 0.50; Copper: 0.50; Zinc: 6.67; Iron: 6.67; Cobalt: 0.50 283; ^2^ Commercial products: Prebiotic: mano-oligosaccharide, Antioxidant: butylhydroxytoluene; ^3^ Price: USD 1 = PEN 3.70.

**Table 6:** Growth and economic performances of trout fed the experimental diets (mean ± standard deviation) first phase.

Item	Experimental Diets			
	Control	PBM 5%	Commercial	*p*-Values
IBW, g	4.17 ± 0.22 ^a^	4.32 ± 0.27 ^a^	4.53 ± 0.41 ^a^	0.307
FBW, g	20.39 ± 0.75 ^a^	21.33 ± 0.59 ^a^	21.61 ± 1.62 ^a^	0.298
WG, g	16.22 ± 0.70 ^a^	17.01 ± 0.53 ^a^	17.09 ± 1.32 ^a^	0.377
Feed intake, (g/fish)	13.13 ± 0.30 ^a^	13.55 ± 0.34 ^a^	13.62 ± 0.77 ^a^	0.385
FCR	0.81 ± 0.02 ^a^	0.80 ± 0.01 ^a^	0.80 ± 0.02 ^a^	0.616
Small size (kg)	35.10 ± 3.01 ^a^	37.80 ± 1.53 ^a^	36.08 ± 4.45 ^a^	0.513
Large size (kg)	43.55 ± 3.87 ^a^	44.38 ± 2.25 ^a^	47.05 ± 3.89 ^a^	0.361
ECR	2.12 ± 0.06 ^a^	2.04 ± 0.04 ^b^	1.86 ± 0.04 ^c^	0.0001

IBW = initial body weight; FBW = final body weight; WG = weight gain; FCR: Feed conversion ratio; ECR = Economic conversion ratio; ^a,b,c^ Different letters indicate significant differences (*p* < 0.05).

**Table 7:** Growth and economic performances of trout fed the experimental diets (mean ± standard deviation) second phase.

Item	Experimental Diets Second Phase A			
	Control	PBM 18%	Commercial	*p*-Value
IBW, g	24.35 ± 0.75 ^a^	24.65 ± 0.87 ^a^	23.84 ± 1.06 ^a^	0.470
FBW, g	58.88 ± 2.36 ^a^	58.47 ± 2.11 ^a^	56.81 ± 0.65 ^a^	0.301
WG, g	34.53 ± 1.69 ^a^	33.82 ± 2.00 ^a^	32.98 ± 0.56 ^a^	0.402
Feed intake, (g/fish)	27.43 ± 0.96 ^a^	27.35 ± 0.60 ^a^	27.28 ± 0.97 ^a^	0.969
FCR	0.79 ± 0.02 ^a^	0.81 ± 0.03 ^a^	0.83 ± 0.04 ^a^	0.310
Small size (kg)	27.33 ± 1.70 ^b^	28.08 ± 1.30 ^b^	33.93 ± 3.28 ^a^	0.005
Large size (kg)	40.00 ± 3.67 ^a^	38.93 ± 1.88 ^a^	31.13 ± 3.17 ^b^	0.005
ECR, USD/kg	1.92 ± 0.04 ^a^	1.76 ± 0.07 ^b^	1.79 ± 0.09 ^b^	0.022
Item	Experimental diets second phase B			
	Control	PBM 18%	Commercial	*p*-value
IBW, g	64.88 ± 2.67 ^a^	62.13 ± 2.90 ^a^	62.99 ± 3.26 ^a^	0.438
FBW, g	128.88 ± 8.35 ^a^	124.50 ± 9.85 ^a^	119.95 ± 7.74 ^a^	0.388
WG, g	64.00 ± 6.97 ^a^	62.38 ± 7.09 ^a^	56.96 ± 4.66 ^a^	0.306
Feed intake, (g/fish)	0.06 ± 2.91 ^a^	0.06 ± 4.17 ^a^	0.06 ± 4.54 ^a^	0.917
FCR	0.91 ± 0.07 ^a^	0.93 ± 0.05 ^a^	1.00 ± 0.03 ^a^	0.118
ECR, USD/kg	2.19 ± 0.18 ^a^	2.02 ± 0.12 ^a^	2.15 ± 0.06 ^a^	0.176

^a,b^ Different letters indicate significant differences (*p* < 0.05).

**Table 8:** Growth and economic performances of trout fed the experimental diets (mean ± standard deviation) third phase.

Item	Experimental Diets			
	Control	PBM 18%	Commercial	*p*-Value
IBW, g	128.88 ± 8.35 ^a^	124.50 ± 9.85 ^a^	119.95 ± 7.74 ^a^	0.388
FBW, g	231.89 ± 14.67 ^a^	229.47 ± 11.90 ^a^	216.58 ± 21.02 ^a^	0.399
WG, g	103.02 ± 8.35 ^a^	104.96 ± 5.51 ^a^	96.63 ± 13.94 ^a^	0.489
Feed intake, (g/fish)	0.10 ± 5.67 ^a^	0.11 ± 6.98 ^a^	0.10 ± 8.07 ^a^	0.542
FCR	1.02 ± 0.04 ^a^	1.02 ± 0.09 ^a^	1.06 ± 0.07 ^a^	0.709
Small size (kg)	13.54 ± 2.92 ^a^	14.94 ± 5.12 ^a^	16.36 ± 3.61 ^a^	0.623
Large size (kg)	79.11 ± 8.51 ^a^	73.78 ± 12.38 ^a^	70.01 ± 11.28 ^a^	0.517
ECR, USD/kg	2.02 ± 0.08 ^a^	1.78 ± 0.15 ^b^	2.05 ± 0.14 ^a^	0.025

^a,b^ Different letters indicate significant differences (*p* < 0.05).

**Table 9:** Proximate composition (% of wet weight) of trout fed the experimental diets replacing various levels of fish meal with poultry by-product meal for 139 days.

Item	Experimental Diets			
	Control	PBM	Commercial	*p*-Value
Dry matter	93.51 ± 0.41 ^a^	93.18 ± 0.43 ^a^	93.36 ± 1.45 ^a^	0.874
Protein	56.62 ± 1.72 ^a^	56.14 ± 1.56 ^a^	57.13 ± 2.79 ^a^	0.874
Lipid	29.66 ± 1.61 ^a^	29.15 ± 1.44 ^a^	29.10 ± 3.34 ^a^	0.804
Ash	5.75 ± 0.43 ^b^	6.39 ± 0.94 ^a^	5.30 ± 0.19 ^b^	0.109

^a,b^ Different letters indicate significant differences (*p* < 0.05).

## Data Availability

https://drive.google.com/file/d/1a5Rv5kFOmrrP-0n4Ej5EqsSw6W8Na0MF/view?usp=sharing (accessed on 12 June 2025).

## Supplementary Materials

The following supporting information can be downloaded at: https://www.mdpi.com/article/10.3390/ani15182723/s1.

## Abbreviations

The following abbreviations are used in this manuscript:

PBM	Poultry by-product meal
FCR	Feed conversion ratio
ECR	Economic conversion ratio
