# Supplementary material for: Substituting Fishmeal with Poultry By-Product Meal Enhances Economic Efficiency in Rainbow Trout (Oncorhynchus mykiss) Farming
Source: Animals (Basel). 2025 Sep 17;15(18):2723. doi: 10.3390/ani15182723 (PMC12466695; doi:10.3390/ani15182723)
Supplement: Supplementary file 1 [file animals-15-02723-s001.zip › animals-3583477-supplementary.pdf]

SUBSTITUTING FISHMEAL WITH POULTRY BY-PRODUCT MEAL ENHANCES ECONOMIC EFFICIENCY IN RAINBOW TROUT (Oncorhynchus FARMING

Table S1. Growth and economic performance of trout fed the experimental diets in the first phase.

	IBW, g	FBW, g	WG, g	Feed intake, (g/fish)	FCR	ECR	Small size (kg)	Large size (kg)
1	4.32	19.91	15.59	12.99	0.83	2.18	39.30	37.60
1	4.40	21.07	16.67	13.48	0.81	2.11	48.70	32.70
1	3.94	19.59	15.65	12.78	0.82	2.13	43.20	32.30
1	4.02	20.99	16.97	13.26	0.78	2.04	43.00	37.80
2	4.66	22.02	17.36	14.01	0.81	2.07	47.60	37.30
2	4.40	20.73	16.33	13.21	0.81	2.07	43.90	36.00
2	4.09	20.95	16.86	13.38	0.79	2.03	42.40	38.30
2	4.12	21.60	17.48	13.61	0.78	1.99	43.60	39.60
3	4.33	22.37	18.04	14.04	0.78	1.82	52.40	33.80
3	4.51	20.67	16.16	13.12	0.81	1.90	44.60	35.10
3	4.17	19.92	15.75	12.84	0.82	1.90	43.80	32.80
3	5.10	23.49	18.39	14.48	0.79	1.84	47.40	42.60

Table S2. Growth and economic performances of trout fed the experimental diets second phase A.

	IBW, g	FBW, g	WG, g	Feed intake, (g/fish)	FCR	ECR	Small size (kg)	Large size (kg)
1	24.54	58.27	33.72	27.55	0.82	1.97	39.60	27.00
1	23.33	56.37	33.04	26.46	0.80	1.93	37.40	27.20
1	24.39	58.83	34.44	27.00	0.78	1.89	37.70	29.60
1	25.13	62.05	36.92	28.70	0.78	1.88	45.30	25.50
2	25.58	58.01	32.43	27.30	0.84	1.82	39.00	27.60
2	23.77	55.67	31.90	26.61	0.83	1.81	36.70	27.10
2	24.06	60.23	36.17	28.09	0.78	1.68	41.30	27.60
2	25.17	59.95	34.77	27.40	0.79	1.71	38.70	30.00
3	25.11	57.76	32.65	28.65	0.88	1.90	29.50	36.40
3	24.16	56.58	32.42	27.23	0.84	1.82	31.00	33.90
3	23.47	56.63	33.16	26.71	0.81	1.74	35.60	29.30
3	22.61	56.28	33.67	26.51	0.79	1.70	28.40	36.10

Table S3. Growth and economic performances of trout fed the experimental diets second phase B.

	IBW, g	FBW, g	WG, g	Feed intake, (g/fish)	FCR	ECR
1	61.50	126.43	64.93	55.04	0.85	2.05
1	66.00	124.53	58.53	56.08	0.96	2.32
1	64.25	123.30	59.05	57.79	0.98	2.36
1	67.75	141.25	73.50	61.67	0.84	2.03
2	59.25	118.05	58.80	53.65	0.91	1.98
2	60.00	114.16	54.16	54.83	1.01	2.19
2	64.75	133.73	68.98	61.60	0.89	1.93
2	64.50	132.08	67.58	61.23	0.91	1.96
3	67.13	129.20	62.07	60.42	0.97	2.10
3	61.60	114.16	52.56	53.74	1.02	2.21
3	59.50	112.99	53.49	51.98	0.97	2.10
3	63.75	123.46	59.71	60.82	1.02	2.20

Table S4. Growth and economic performances of trout fed the experimental diets third phase

	IBW, g	FBW, g	WG, g	Feed intake, (g/fish)	FCR	ECR	Small size (kg)	Large size (kg)
1	126.43	217.94	91.52	98.53	1.08	2.14	72.46	14.50
1	124.53	230.90	106.38	105.53	0.99	1.97	77.39	14.97
1	123.30	226.35	103.05	102.92	1.00	1.98	75.07	15.47
1	141.25	252.38	111.13	112.09	1.01	2.00	91.50	9.20
2	118.05	229.40	111.35	100.09	0.90	1.57	59.30	20.90
2	114.16	213.16	99.00	101.63	1.03	1.79	67.65	17.40
2	133.73	241.18	107.46	113.64	1.06	1.84	84.32	11.67
2	132.08	234.13	102.05	112.09	1.10	1.91	83.86	9.79
3	129.20	234.09	104.89	108.86	1.04	2.02	76.00	17.40
3	114.16	199.75	85.59	93.88	1.10	2.13	62.10	17.60
3	112.99	197.06	84.07	94.70	1.13	2.19	59.10	19.33
3	123.46	235.41	111.95	107.61	0.96	1.87	82.83	11.10

Table S5. Performances of trout fed the experimental diets in the first phase.

**Item**	**Experimental diets**			**P-values**	[1]**Pr > ChiSq**
	**Control**	**PBM 5%**	**Commercial**		
IBW, g	4.17 ± 0.22 [a]	4.32 ± 0.27 [a]	4.53 ± 0.41 [a]	0.307	0.6035
FBW, g	20.39 ± 0.75 [a]	21.33 ± 0.59 [a]	21.61 ± 1.62 [a]	0.298	0.2234
WG, %	16.22 ± 0.70 [a]	17.01 ± 0.53 [a]	17.09 ± 1.32 [a]	0.377	0.3050
Feed intake, (g/fish)	13.13 ± 0.30 [a]	13.55 ± 0.34 [a]	13.62 ± 0.77 [a]	0.385	0.2482
FCR	0.81 ± 0.02 [a]	0.80 ± 0.01 [a]	0.80 ± 0.02 [a]	0.616	0.8431
Small size (kg)	35.10 ± 3.01 [a]	37.80 ± 1.53 [a]	36.08 ± 4.45 [a]	0.513	0.63983
Large size (kg)	43.55 ± 3.87 [a]	44.38 ± 2.25 [a]	47.05 ± 3.89 [a]	0.361	0.2741
ECR	2.12 ± 0.06 [a]	2.04 ± 0.04 [b]	1.86 ± 0.04 [c]	0.000	0.7627

[1]Bartlett's test for homogeneity of variances was not significant for the evaluated parameters, therefore homogeneity of variances can be assumed.

Table S6. Normality of residuals of the first phase

Pr < W	0.3248

The Shapiro-Wilk normality test was not significant, indicating that the residuals are normal.

Figure S1. Normality

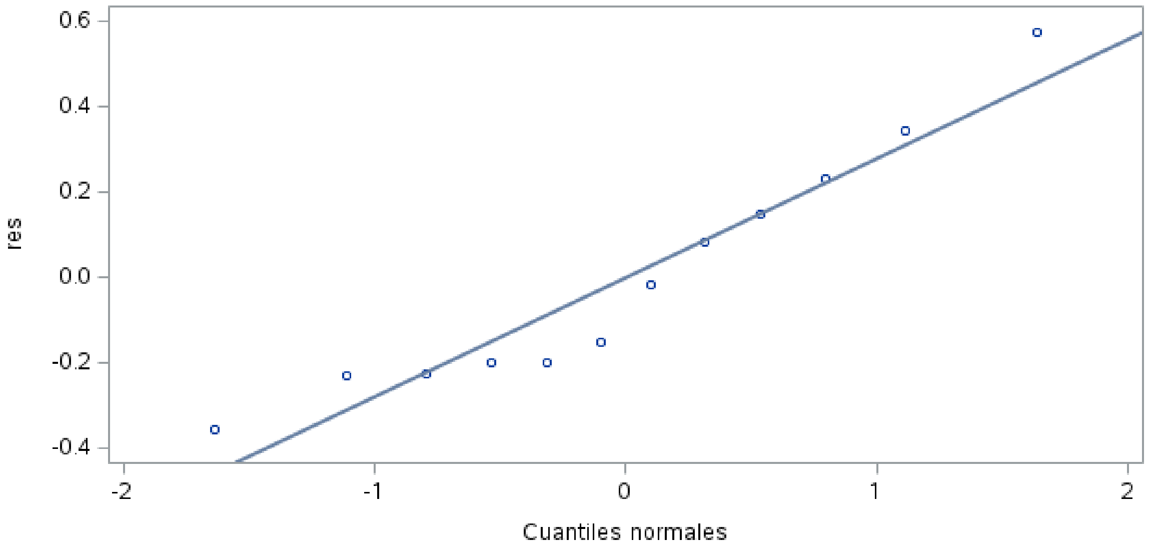

Figure S2. independence of errors

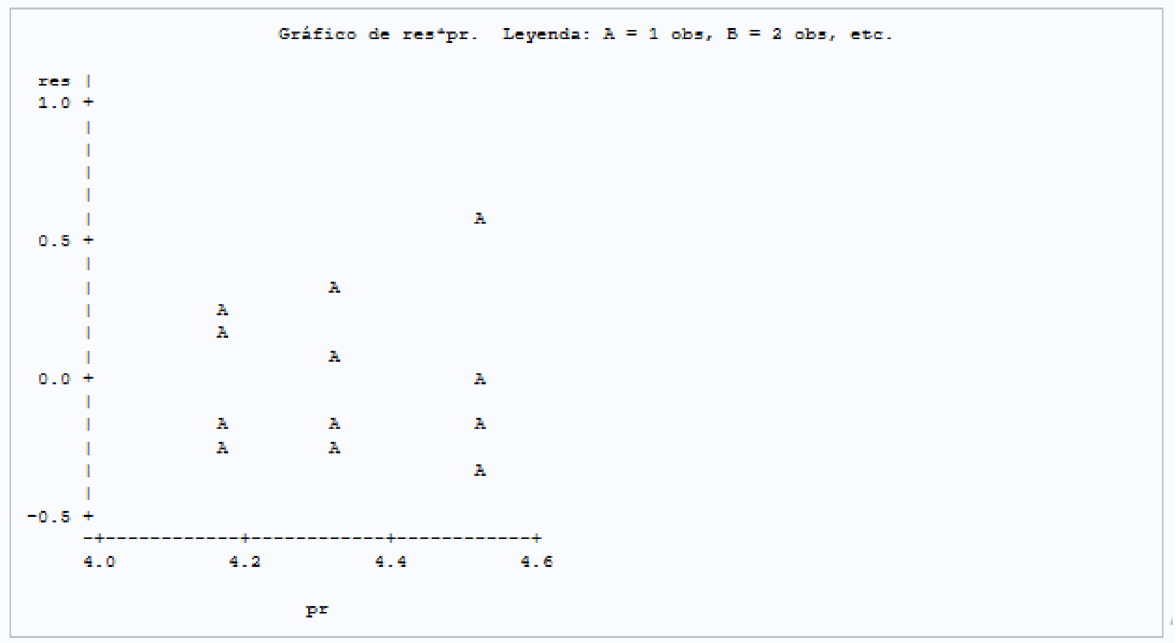

Since the plot shows no discernible trend, we can infer independence of errors

Table S7. Performances of trout fed the experimental diets second phase A.

**Item**	**Experimental diets second phase A**			**P-values**	[2]**Pr > ChiSq**
	**Control**	**PBM 18%**	**Commercial**		
IBW, g	24.35 ± 0.75 ^{a}	24.65 ± 0.87 ^{a}	23.84 ± 1.06 ^{a}	0.470	0.8536
FBW, g	58.88 ± 2.36 ^{a}	58.47 ± 2.11 ^{a}	56.81 ± 0.65 ^{a}	0.301	0.1621
WG, %	34.53 ± 1.69 ^{a}	33.82 ± 2.00 ^{a}	32.98 ± 0.56 ^{a}	0.402	0.1714
Feed intake, (g/fish)	27.43 ± 0.96 ^{a}	27.35 ± 0.60 ^{a}	27.28 ± 0.97 ^{a}	0.969	0.7201
FCR	0.79 ± 0.02 ^{a}	0.81 ± 0.03 ^{a}	0.83 ± 0.04 ^{a}	0.310	0.5369
Small size (kg)	27.33 ± 1.70 ^{b}	28.08 ± 1.30 ^{b}	33.93 ± 3.28 ^{a}	0.005	0.5754
Large size (kg)	40.00 ± 3.67 ^{a}	38.93 ± 1.88 ^{a}	31.13 ± 3.17 ^{b}	0.005	0.2968
ECR, USD/kg	1.92 ± 0.04 ^{a}	1.76 ± 0.07 ^{b}	1.79 ± 0.09 ^{b}	0.022	0.4934

[1]Bartlett's test for homogeneity of variances was not significant for the evaluated parameters, therefore homogeneity of variances can be assumed.

Table S8. Normality of residuals of the first phase

Pr < W	0.8085

The Shapiro-Wilk normality test was not significant, indicating that the residuals are normal.

Figure S3. Normality

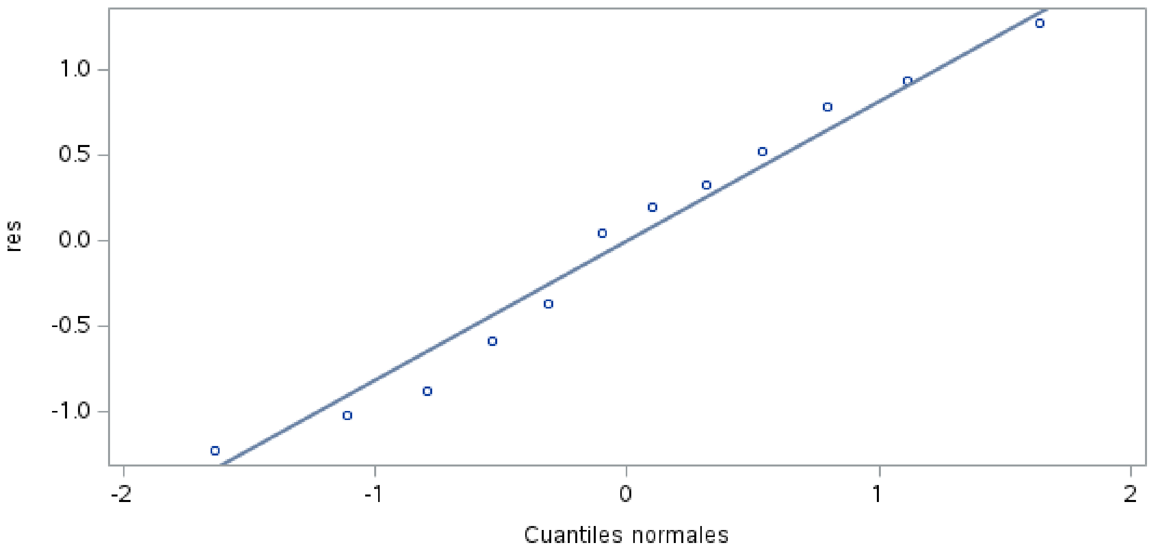

Figure S4. independence of errors

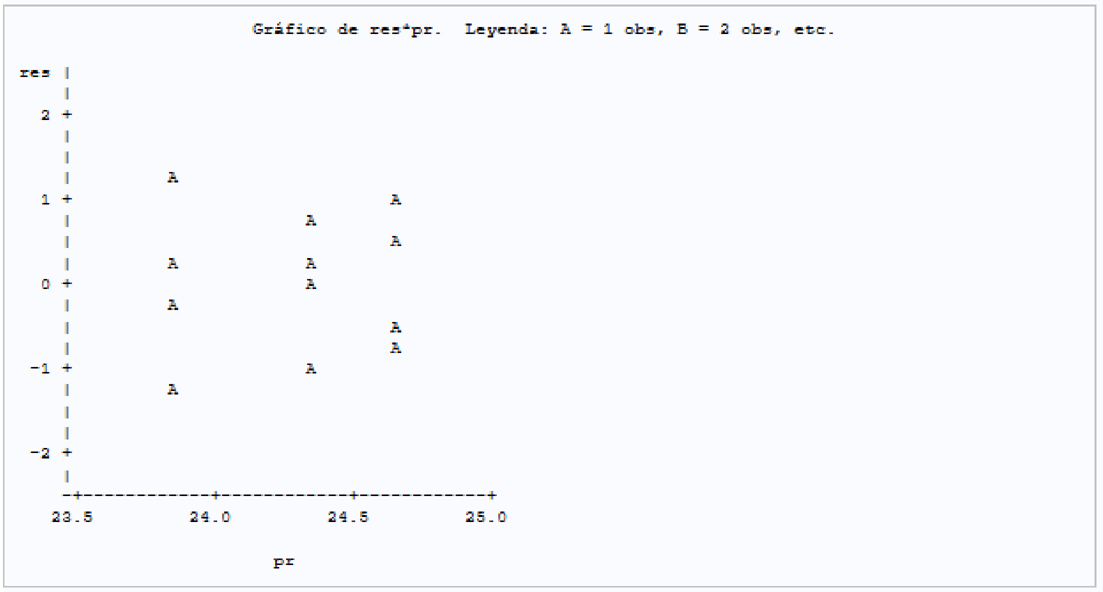

Since the plot shows no discernible trend, we can infer independence of errors

Table S9. Performances of trout fed the experimental diets second phase B.

**Item**	**Experimental diets second phase B**			**P-values**	**[1]Pr > ChiSq**
	**Control**	**PBM 18%**	**Commercial**		
IBW, g	64.88 ± 2.67 [a]	62.13 ± 2.90 [a]	62.99 ± 3.26 [a]	0.438	0.9482
FBW, g	128.88 ± 8.35 [a]	124.50 ± 9.85 [a]	119.95 ± 7.74 [a]	0.388	0.9220
WG, %	64.00 ± 6.97 [a]	62.38 ± 7.09 [a]	56.96 ± 4.66 [a]	0.306	0.7674
Feed intake, (g/fish)	0.06 ± 2.91 [a]	0.06 ± 4.17 [a]	0.06 ± 4.54 [a]	0.917	0.7682
FCR	0.91 ± 0.07 [a]	0.93 ± 0.05 [a]	1.00 ± 0.03 [a]	0.118	0.3728
ECR, USD/kg	2.19 ± 0.18 [a]	2.02 ± 0.12 [a]	2.15 ± 0.06 [a]	0.176	0.2847

[1]Bartlett's test for homogeneity of variances was not significant for the evaluated parameters, therefore homogeneity of variances can be assumed.

Table S10. Normality of residuals of the first phase

Pr < W	0.3650

The Shapiro-Wilk normality test was not significant, indicating that the residuals are normal.

Figure S5. Normality

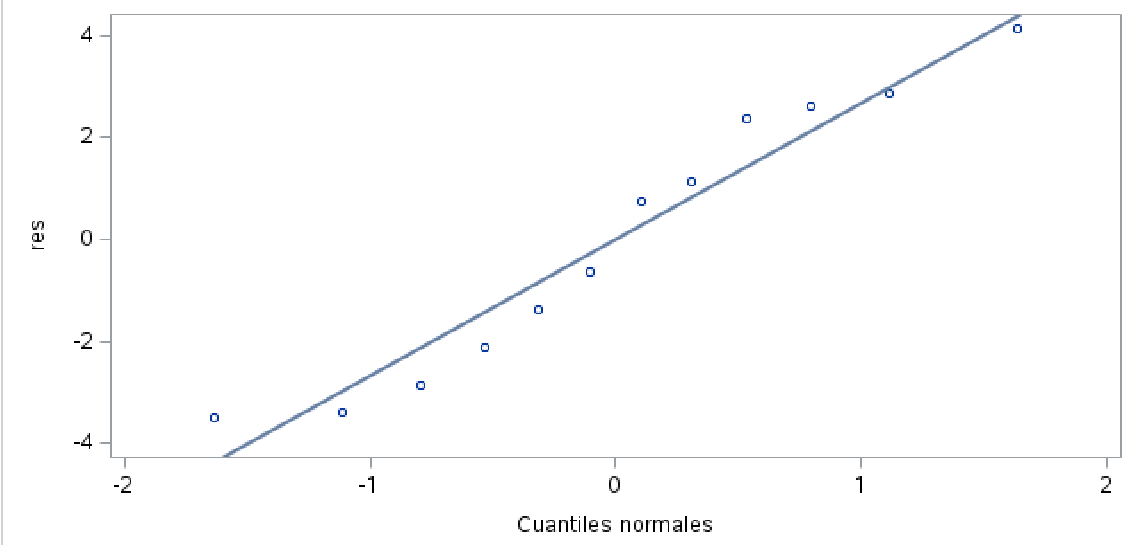

Figure S6. Independence of errors

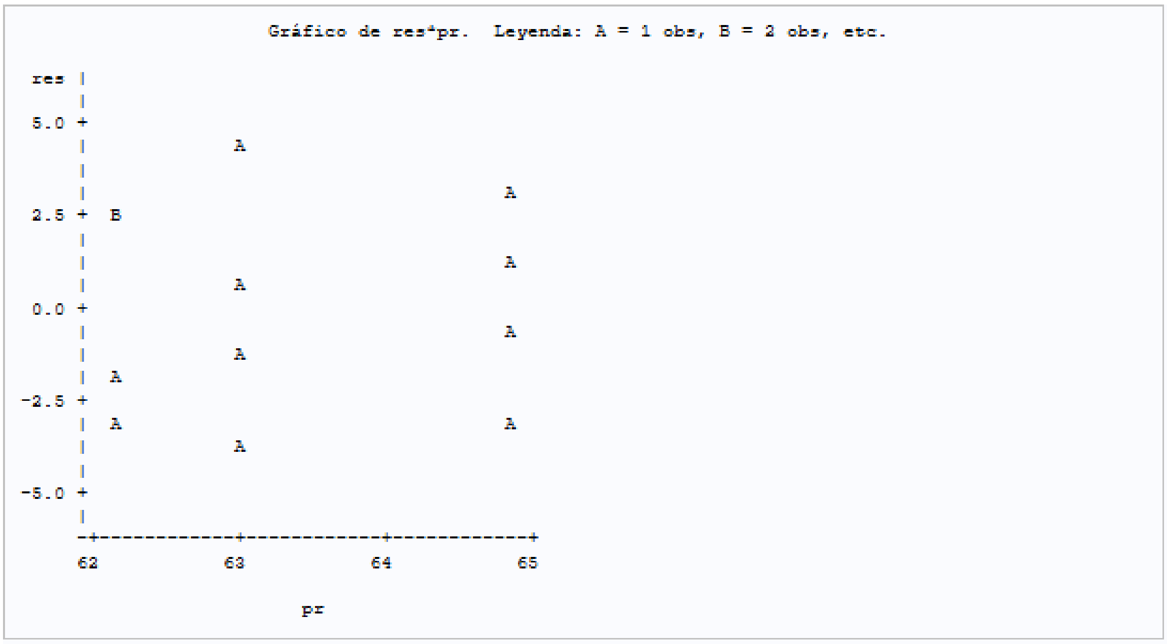

Since the plot shows no discernible trend, we can infer independence of errors

Table S11. Performances of trout fed the experimental diets third phase.

**Item**	**Experimental diets third phase**			**P-values**	**[1]Pr > ChiSq**
	**Control**	**PBM 18%**	**Commercial**		
IBW, g	128.88 ± 8.35 ^{a}	124.50 ± 9.85 ^{a}	119.95 ± 7.74 ^{a}	0.388	0.9220
FBW, g	231.89 ± 14.67 ^{a}	229.47 ± 11.90 ^{a}	216.58 ± 21.02 ^{a}	0.399	0.6438
WG, %	103.02 ± 8.35 ^{a}	104.96 ± 5.51 ^{a}	96.63 ± 13.94 ^{a}	0.489	0.3377
Feed intake, (g/fish)	0.10 ± 5.67 ^{a}	0.11 ± 6.98 ^{a}	0.10 ± 8.07 ^{a}	0.542	0.8525
FCR	1.02 ± 0.04 ^{a}	1.02 ± 0.09 ^{a}	1.06 ± 0.07 ^{a}	0.709	0.4985
Small size (kg)	13.54 ± 2.92 ^{a}	14.94 ± 5.12 ^{a}	16.36 ± 3.61 ^{a}	0.623	0.8298
Large size (kg)	79.11 ± 8.51 ^{a}	73.78 ± 12.38 ^{a}	70.01 ± 11.28 ^{a}	0.517	0.6508
ECR, USD/kg	2.02 ± 0.08 ^{a}	1.78 ± 0.15 ^{b}	2.05 ± 0.14 ^{a}	0.025	0.5933

[1]Bartlett's test for homogeneity of variances was not significant for the evaluated parameters, therefore homogeneity of variances can be assumed.

Table S12. Normality of residuals of the first phase

Pr < W	0.1000

The Shapiro-Wilk normality test was not significant, indicating that the residuals are normal.

Figure S7. Normality

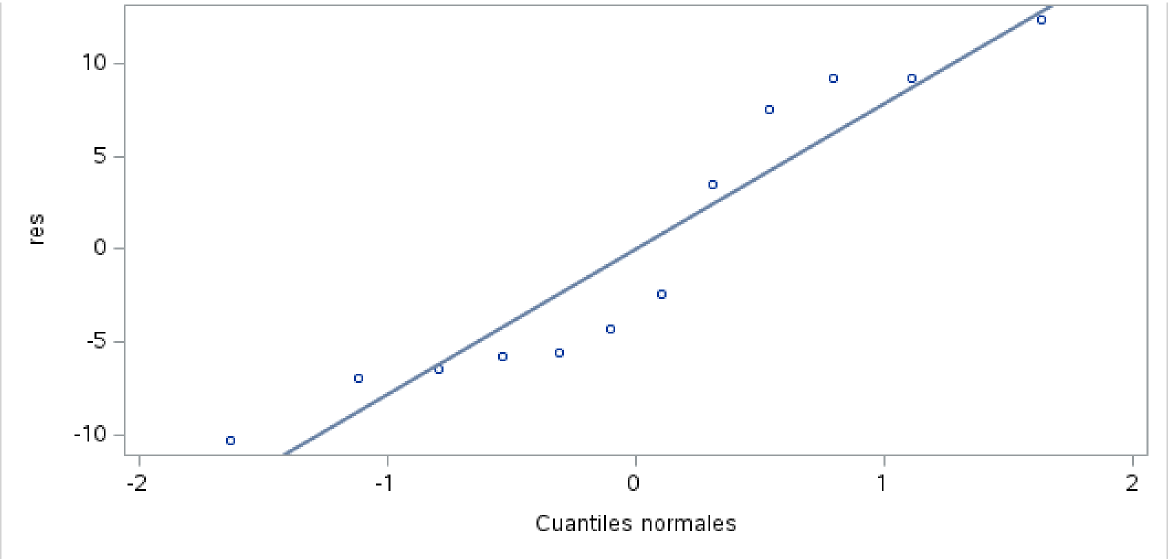

Figure S8. Independence of errors

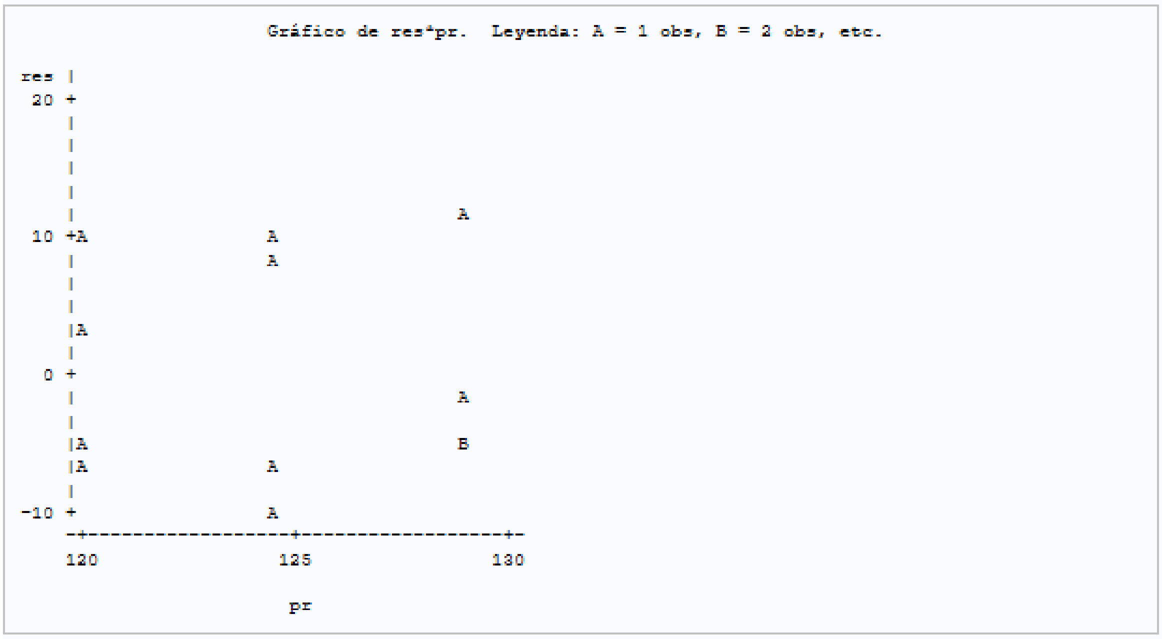

Since the plot shows no discernible trend, we can infer independence of errors
